# Competing Endogenous RNA Regulations in Neurodegenerative Disorders: Current Challenges and Emerging Insights

**DOI:** 10.3389/fnmol.2018.00370

**Published:** 2018-10-05

**Authors:** Yifei Cai, Jun Wan

**Affiliations:** ^1^Shenzhen Key Laboratory for Neuronal Structural Biology, Biomedical Research Institute, Shenzhen Peking University – The Hong Kong University of Science and Technology Medical Center, Shenzhen, China; ^2^Division of Life Science, The Hong Kong University of Science and Technology, Clear Water Bay, Hong Kong

**Keywords:** competing endogenous RNA (ceRNA), neurodegenerative disorder, miRNA hierarchy, miRNA stabilization, ceRNA debate

## Abstract

The past decade has witnessed exciting breakthroughs that have contributed to the richness and complexity of a burgeoning modern RNA world, and one particular breakthrough—the competing endogenous RNA (ceRNA) hypothesis—has been described as the “Rosetta Stone” for decoding the RNA language used in regulating RNA crosstalk and modulating biological functions. The proposed far-reaching mechanism unites diverse RNA species and provides new insights into previously unrecognized RNA–RNA interactions and RNA regulatory networks that perhaps determine gene expression in an organized, hierarchical manner. The recently uncovered ceRNA regulatory loops and networks have emphasized the power of ceRNA regulation in a wide range of developmental stages and pathological contexts, such as in tumorigenesis and neurodegenerative disorders. Although the ceRNA hypothesis drastically enhanced our understanding of RNA biology, shortly after the hypothesis was proposed, disputes arose in relation mainly to minor discrepancies in the reported effects of ceRNA regulation under physiological conditions, and this resulted in ceRNA regulation becoming an extensively studied and fast-growing research field. Here, we focus on the evidence supporting ceRNA regulation in neurodegenerative disorders and address three critical points related to the ceRNA regulatory mechanism: the microRNA (miRNA) and ceRNA hierarchies in cross-regulations; the balance between destabilization and stable binding in ceRNA–miRNA interactions; and the true extent to which ceRNA regulatory mechanisms are involved in both health and disease, and the experimental shortcomings in current ceRNA studies.

## A Rising Modern RNA World

Following in the footsteps of the Human Genome Project and the next-generation sequencing of model organisms, myriad ncRNAs were identified, such as miRNA (Lee et al., 1993), siRNA (Waterhouse et al., 1998), lncRNA (Okazaki et al., 2002), and circRNA (Capel et al., 1993). ENCODE reported that 80% of the human genome is transcribed as non-coding transcripts (Djebali et al., 2012; Rosenbloom et al., 2012), and these manipulate 80% of the DNA with potential biological functions (Consortium, 2012). Studies conducted over the past two decades have shown that ncRNAs are widely expressed in specific spatiotemporal patterns in various species and are extensively involved in numerous biological processes, including epigenetic regulation, chromatin remodeling, transcription control, and posttranscriptional processing (Eddy, 2001). Progressively increasing numbers of ncRNAs were identified to play important roles in modulating NDDs pathogenesis. In this session, we briefly introduce the characteristics and functions of lncRNAs, miRNAs, and circRNAs, which are three main types of RNAs reported in the ceRNA regulations in NDDs.

lncRNA is an RNA transcript containing > 200 nt that generates no protein product (Okazaki et al., 2002). lncRNAs are typically 5′-capped and polyadenylated (Quinn and Chang, 2016). lncBase had annotated > 56,000 lncRNA transcripts thus far (Paraskevopoulou et al., 2013; Necsulea et al., 2014; Cao et al., 2018). lncRNAs can be transcribed from multiple genomic structures, such as exons, introns, promoters, 3′ UTRs, and intergenic regions, and the transcription direction can be sense or antisense or both (Mercer et al., 2009). Recent studies have reported the presence of hidden ORFs in a few lncRNAs, and thus small peptides could be translated in these cases and perform biological functions (Anderson et al., 2015; Makarewich and Olson, 2017); this blurs the boundary between lncRNAs and mRNAs to certain extent. lncRNAs participate in modulating nervous system in various biological dimensions, such as through epigenetic regulation (Mercer and Mattick, 2013), or transcriptional and posttranscriptional regulation (Mercer et al., 2009). For example, β-site amyloid precursor protein (APP)-cleaving enzyme 1-antisense (BACE1-AS) stabilizes BACE1 RNA and promotes APP cleavage, and is actively involved in AD pathogenesis (Faghihi et al., 2008; Esteller, 2011). Evf2 regulates adult hippocampal GABA neural circuits by controlling downstream target gene-expression dynamics (Bond et al., 2009). Both HOTAIR (Esteller, 2011) and MALAT1 (Tripathi et al., 2010) are upregulated in and promote brain tumor metastasis, and Malat1 regulates synaptogenesis in mouse hippocampal neurons by controlling gene expression (Bernard et al., 2010).

miRNAs are 22-nt-long ncRNAs that induce target-RNA silencing or degradation through complementary base-pairing with the miRNA response elements (MREs) on their 3′ UTRs by interacting with RISC (Guil and Esteller, 2015). There are 519 confidently identified canonical miRNA genes in the human genome (Denzler et al., 2014; Fromm et al., 2015; Bartel, 2018). Roughly 70% of the identified miRNAs are expressed in the brain and in neurons (Cao et al., 2006), and miRNAs thus perform critical regulatory functions in central nervous system (CNS) development, dendritic spine formation, neurite outgrowth, and neuronal differentiation and maintenance. Moreover, miRNA deregulations are involved in NDDs such as AD and PD (Esteller, 2011), as well as in psychiatric disorders such as schizophrenia (Xu et al., 2013). Multiple lines of evidence indicate that miRNAs are associated with AD; for example, let-7, miR-15a, and miR-101 target APP, whereas miR-15a, miR-9, and miR-107 regulate BACE1 (De Smaele et al., 2010).

circRNAs can be formed either through direct covalent joining of the 5′ and 3′ ends of a linear RNA, such as from an intron, or in a “backsplicing” manner, where a downstream 5′ splice donor site is joined to an upstream 3′ splice acceptor site, generating a circular transcript (Lasda and Parker, 2014). Because of forming a covalent closed-loop structure that lacks 5′ and 3′ ends, circRNAs are more resistant to exonucleases and more stable in cells than linear RNAs (Lasda and Parker, 2014; Zhang et al., 2017). Experimental results have shown that circRNAs form a large class of posttranscriptional regulators. circRNAs containing MREs shared by linear ncRNAs might enable and modulate ceRNA crosstalk and ceRNA networks by reducing miRNA pressure on protein-coding RNAs in a tissue- or cell-specific manner, thereby acting as circ-ceRNAs (Taulli et al., 2013), which will be further discussed in the next session. The potential functions of the circRNAs remain to be identified; for example, circRNAs could (1) represent a powerful weapon against endogenous miRNAs and enhance ceRNA crosstalk (Taulli et al., 2013); (2) act as binding and storage components that sort and deliver factors to specific subcellular locations; or (3) function as scaffolds for the assembly of other complexes or reactions (Lasda and Parker, 2014). circRNAs play critical roles in maintaining nervous system functions, and most circRNAs exhibit an expression pattern that is associated with specific neuroanatomical regions, cell types, or subcellular compartments (Chen et al., 2016). You et al. (2015) reported that a disproportionately high fraction of circRNA is derived from synaptic protein-coding genes and enriched in synaptic tissues. The expression levels of these brain-enriched circRNAs are regulated in a manner that is correlated with neural plasticity and developmental stages (You et al., 2015). Moreover, circRNA expression was reported to increase relative to that of linear mRNAs during aging (Westholm et al., 2014; Ivanov et al., 2015), and emerging evidence indicates that the circRNA-ceRNA machinery is actively involved in the pathogenesis of NDDs (Shao and Chen, 2016), as we discuss in the next two sections.

## Competing Endogenous RNA Hypothesis: RNA Crosstalk

**FIGURE 1:**
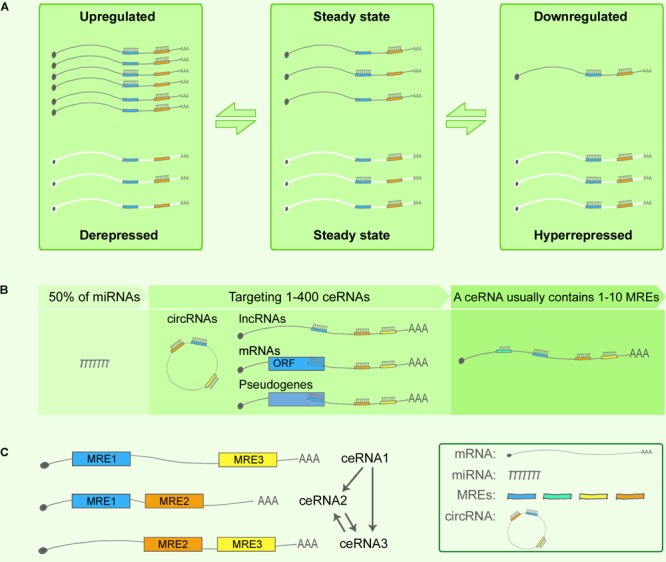
Competing endogenous RNA theory. **(A)** ceRNA crosstalk. Upregulation of ceRNA X (black) expression increases cellular concentrations of specific MREs and can result in the de-repression of other transcripts (ceRNA Y, white) that contain the same MREs. Conversely, downregulation of ceRNA X would lead to decreased concentrations of specific MREs and thus to over-repression of ceRNA Y expression. **(B)** ceRNA targets. Predictions from TargetScan database revealed that 50% of all miRNAs target 1–400 mRNAs, and that most ceRNAs contain 1–10 MREs. **(C)** Indirect interaction within a simplified ceRNET.

In 2011, Pier Paolo Pandolfi’s group proposed the ceRNA hypothesis, positing that RNAs can engage in crosstalk with each other and manipulate biological functions independently of protein translation (Salmena et al., 2011). The foundation of the ceRNA hypothesis is the miRNA-RISC-directed posttranscriptional silencing or degradation of target RNAs (Gregory et al., 2005), as noted in an earlier section, and in these biological processes, the complementary base-pairing of 6–8-nt-long MREs serves as the language for RNA communication and RNA network regulation. **Figure 1A** shows the most simplified ceRNA model: ceRNAs X and Y share mutual miRNAs, and when ceRNA X is upregulated, it can compete for binding with an increased amount of miRNAs and lead to ceRNA Y de-repression; conversely, when ceRNA X is downregulated, more miRNAs can bind with ceRNA Y and thereby cause ceRNA Y over-repression. Thus, a positive correlation exists between the expression levels of ceRNA X and Y (Salmena et al., 2011). A network can include various type of ceRNAs, such as lncRNAs, circRNAs, pseudogenes, and mRNAs (Fatica and Bozzoni, 2014) (**Figure 1B**).

The network regulation of ceRNAs can be complex. Based on the predictions from TargetScan database, 50% of all miRNAs target 1–400 mRNAs, and certain rare miRNAs can target > 1,000 mRNAs. Similarly, according to TargetScan predictions, most ceRNAs contain 1–10 MREs (Ala et al., 2013) (**Figure 1B**). Consequently, numerous ceRNA–miRNA interactions generate highly complex ceRNA networks (ceRNETs). In 2013, the Pandolfi group attempted to decode how single-factor perturbations influence ceRNETs and how these innate factors contribute synergistically to the amplification of downstream signaling cascades. First, ceRNET efficacy could be maximized when ceRNA and miRNA expression levels are below a certain optimized threshold, whereas perturbations of miRNA levels can disrupt the ceRNET balance; second, the higher the number of MREs in a ceRNA, the higher the efficacy achieved by the ceRNET; and third, indirect ceRNA crosstalk plays critical roles in amplifying the downstream signaling (Ala et al., 2013) (**Figure 1C**).

## Competing Endogenous RNA in Neurodegenerative Disorders

Over the last 7 years, the ceRNA hypothesis has been validated by the results of numerous experiments. However, ceRNA mechanisms and network construction have thus far been mainly studied in the field of cancer research (Poliseno et al., 2010; Sumazin et al., 2011; Tay et al., 2011; Taulli et al., 2013; Karreth et al., 2015). Only a few ceRNA interactions have been reported in NDDs in the past 10 years, and researchers have started to explore the ceRNA regulatory mechanism in NDDs by cases and in a systematic manner only during the last 2 years. Nevertheless, exciting advances have been made in our understanding of ceRNA interactions in NDDs. In this section, we categorize these ceRNA studies to introduce them in the context of distinct NDDs.

NDDs, including AD, PD, amyotrophic lateral sclerosis (ALS), and Huntington’s disease (HD), which produce symptoms such as progressive deficits in neuronal function or structure, typically lead to sustained neuronal death (Johnson et al., 2012). NDDs are highly complex diseases for which effective drugs and treatments are still lacking (Honig et al., 2018). The main causes that have been reported for NDDs are oxidative stress, abnormal protein oligomerization and aggregation, axonal-transport deficits, mitochondrial dysfunction, excitotoxicity and calcium dysregulation, neuronal-glial interactions and neuroinflammation, DNA damage, and aberrant RNA processing (Johnson et al., 2012).

### Alzheimer’s Disease

Alzheimer’s disease, a heterogeneous NDD, was first described by the German psychiatrist and pathologist Alois Alzheimer in 1907 (Selkoe, 2001). The main symptoms of AD are cognitive impairment, mood and behavioral changes, and psychological symptoms such as depression (Karch et al., 2014). AD is categorized as familial AD (FAD) and sporadic AD (SAD). SAD, also known as late-onset AD, accounts for 90% of AD incidence. FAD is reported to associate with inherited mutations in APP, presenilin-1 (PSEN1), and presenilin-2 (PSEN2) (Bekris et al., 2010). AD presents two pathological hallmarks: amyloid-β (Aβ)-induced amyloid plaques (Hardy and Selkoe, 2002; Mattson, 2004; Karch et al., 2014), and neurofibrillary tangles (NFTs) induced by hyperphosphorylated tau protein (Ballatore et al., 2007).

Among the four NDDs mentioned above, AD is the disease in the context of which ceRNAs have been most well studied. The proteolytic processing of APP is catalyzed by BACE1 and this generates amyloid plaques on neurons (Modarresi et al., 2011; Wan et al., 2017). Faghihi et al. (2008) reported BACE1-AS, a conserved antisense transcript of BACE1, which is the first regulatory ceRNA described in AD pathogenesis; BACE1-AS was shown to upregulate the BACE1 mRNA level by forming a stabilizing duplex with it and thereby enhancing BACE1 protein levels. Subsequent work from this group demonstrated that BACE1-AS regulates BACE1 mRNA levels by masking the binding site for miR-485-5p (Faghihi et al., 2010; Roberts et al., 2014), i.e., by functioning as a ceRNA.

Jørgen Kjems and coworkers were among the first researchers to report that circRNAs function as miRNA sponges (Salta and De Strooper, 2017). Hansen et al. first reported that a circular antisense transcript of the CDR1 locus is highly expressed in the human and mouse brain, and that miR-671 directs the cleavage of this circRNA in an Ago2-slicer-dependent manner (Hansen et al., 2011). In addition to stabilizing CDR1 mRNA, the circRNA acts as a miR-7 sponge, and therefore it was also named ciRS-7 (circular RNA sponge for miR-7); ciRS-7 contains > 74 seed-sequence matches for miR-7. In the mouse brain, ciRS-7 and miR-7 are coexpressed specifically in neocortical and hippocampal neurons, which suggests a high degree of endogenous interaction between them (Hansen et al., 2013; Memczak et al., 2013; Salta and De Strooper, 2017). In AD patients, ciRS-7 was found to be downregulated in the hippocampus and cortex, and this was associated with the de-repression of miR-7 and led to the over-repression of the miR-7 downstream target UBE2A, which is involved in ubiquitin-mediated Aβ clearance. Notably, this ciRS-7-miRNA-7-UBE2A circuit is markedly misregulated in the SAD neocortex (Zhao et al., 2016). Moreover, Shi et al. (2017) reported that ciRS-7 plays a protective role in AD by reducing APP and BACE1 protein levels by promoting their degradation through the proteasome and lysosome pathways.

Neurovascular dysfunction participates in AD pathogenesis by influencing Aβ clearance and increasing Aβ levels in the brain (Tanzi et al., 2004). Jiang et al. (2016) showed that the lncRNA MIAT is involved in the maintenance of proper microvascular and nervous function and acts as a ceRNA and regulates VEGF levels by sponging miR-150-5p in retinal endothelial cells. Jiang et al. (2016) also identified a role of MIAT in vasculo-neuronal dysfunction in AD by knocking down MIAT in APP/PSEN mice, which resulted in a substantial reduction of hippocampal SMI-311-positive neurofilaments and markedly diminished GFAP and CD11b/c immunoreactivity in the hippocampus. Whereas MIAT knockdown increased Aβ40 and Aβ42 levels *in vivo*, it strongly downregulated the expression of LRP1, a key Aβ clearance transporter at the blood-brain barrier (Jiang et al., 2016).

The year 2017 witnessed a notable increase in the publication of studies identifying genome-wide ceRNA networks in AD based on the use of distinct disease models (Cai et al., 2017; Wang L.K. et al., 2017; Wang P. et al., 2017; Zhang et al., 2017); this contributed substantially toward a systematic and comprehensive elucidation of ceRNA regulation in AD. Cai et al. (2017) identified one of the first AD-associated lncRNA–miRNA–mRNA networks based on the APP/PSEN1 mouse model, which is a widely used FAD model; whole-transcriptome sequencing and miRNA-seq of the APP/PSEN1 and wild-type mouse cortex were leveraged to identify a ceRNA network that includes 4 hub lncRNAs, 5 miRNAs, and 1,082 mRNA targets. The target mRNAs were significantly enriched in 9 AD-related KEGG pathways, such as “regulation of actin cytoskeleton” and “MAPK signaling” pathways, as well as in an AD-related gene pool (Kim and Choi, 2010; Munoz and Ammit, 2010; Penzes and Vanleeuwen, 2011). Among the miRNAs, miR-326-3p was found to associate significantly with “regulation of actin cytoskeleton” by targeting 4 downstream mRNA targets synergistically, and miR-330-5p shared similar mRNA targets, although it did not show significant association. The lncRNA Rpph1 was upregulated in the APP/PSEN1 mouse cortex, and Cdc42 was upregulated in both the APP/PSEN1 mouse cortex and the hippocampal neurons of AD patients (Zhu et al., 2000). Rpph1 was shown to upregulate Cdc42 RNA and protein levels and promote the formation of hippocampal dendritic spines by targeting miR-330-5p and miR-326-3p, which represents a potential molecular mechanism of synaptic compensation in the early stage of AD pathogenesis (Scheff et al., 1990; Scheff and Price, 1993; Scheff, 2003; Small, 2004; Penzes and Vanleeuwen, 2011; Guo et al., 2013; Abuhassan et al., 2014).

Intracellular NFTs are composed of a highly aggregated and hyperphosphorylated form of the microtubule-associated protein tau, and the formation of these NFTs leads to an impaired dendritic structure, neuronal toxicity, and cell death (Ittner et al., 2010; Ittner and Gotz, 2011). Wang and coworkers constructed an AD-associated lncRNA–miRNA–mRNA triple network based on the hyperphosphorylated tau theory by using 10 matched samples of NFT-harboring and normal neurons from the entorhinal cortex of mid-stage AD cases (Wang L.K. et al., 2017); by mapping the differentially expressed lncRNAs and mRNAs back to a global ceRNA network, the researchers constructed an AD NFT-associated ceRNA network containing 41 lncRNAs, 630 mRNAs, and 2,530 edges. Three AD NFT-associated lncRNAs (AP000265.1, KB-1460A1.5, and RP11-145M9.4) and two AD NFT-related modules were identified. Among the lncRNAs, KB-1460A1.5 was found to be targeted by miR-520 and miR-302 family members, and this was reported to inhibit PTEN, activate Akt, and subsequently inhibit Aβ-induced neurotoxicity. The two modules were found to be enriched in pathways such as protein amino acid phosphorylation and JNK and MAPK cascades, which contribute to AD pathogenesis by regulating APP processing and tau phosphorylation (Kim and Choi, 2010; Munoz and Ammit, 2010). This study provided insights into the molecular basis of ceRNA regulation from the perspective of AD NFTs.

Zhang et al. (2017) elucidated a circRNA-associated ceRNA network based on the senescence-associated mouse prone 8 (SAMP8) model, which shows age-related learning and memory deficits and is used as a mouse model of SAD; as a control strain, Zhang et al. (2017) used senescence-accelerated mouse resistant 1 (SAMR1) mice. AD-associated ceRNA pairs were selected and included 235 circRNAs, 30 miRNAs, and 1,202 mRNAs; these were differentially expressed according to the RNA-seq data and were selected based on two strategies: circRNA (up) – miRNA (down) – mRNA (up), and circRNA (down) – miRNA (up) – mRNA (down). For example, 6 differentially expressed circRNAs targeted Hmgb2 through mmu-let-7g-3p. Hmgb2 was reported to regulate LRP1 expression and contribute to Aβ clearance (Martiskainen et al., 2013; Yamanaka et al., 2015). Another example is that of 5 differentially expressed circRNAs that were predicted to target mmu-miR-122-5p and regulate the downstream target Dio2, which is downregulated in AD patients and contributes to myelination (Calza et al., 2002; Zhan et al., 2014).

DisLncPri, a disease-lncRNA prioritization method, was created to identify unknown disease-lncRNA associations based on a ceRNA theory. Among the top 20 AD-associated lncRNAs, 3 previously unrecognized lncRNAs were identified: MEG3, PVT1, and LINC01616 (Wang P. et al., 2017). All these efforts devoted toward discovering various types of ceRNA regulation in AD not only enhance our understanding of newly identified aspects of ceRNA regulatory mechanisms, but also provide new insights to the complexity of AD pathogenesis.

### Parkinson’s Disease

Parkinson’s disease, the second most common NDD after AD (de Lau and Breteler, 2006), is characterized by severe motor symptoms (Lotharius and Brundin, 2002). The main pathological hallmark of PD is a preferential loss of dopamine-producing neurons and leads to a pronounced depletion of dopamine in the striatum, to which the neurons project. Multiple etiological triggers are linked to PD, such as genetic mutations in α-synuclein and Parkin (Lotharius and Brundin, 2002). The widely expressed intracellular protein α-synuclein triggers aberrant nerve signals, impairing movement coordination and cognition (Saraiva et al., 2016).

The gene *GBA* encodes the enzyme glucocerebrosidase (GCase), which catalyzes the hydrolysis of membrane glucosylceramide (GlcCer) to ceramide and glucose. *GBA* mutations are reported to be strongly associated with PD progression and survival (Cilia et al., 2016). Moreover, GlcCer accumulation directly affects the abnormal lysosomal storage of α-synuclein oligomers in neurons and in the brains of PD patients, which leads to further inhibition of GCase activity, and these bidirectional effects of GlcCer and α-synuclein accumulation could create a positive-feedback loop leading to a self-propagating disease (Mazzulli et al., 2011). Intriguingly, *GBAP1*, a highly homologous (96% sequence identity) and expressed *GBA* pseudogene, is located 16 kb downstream of the functional *GBA* gene (Horowitz et al., 1989; Imai et al., 1993). Straniero et al. (2017) checked for the existence of a *GBA*/miR-22-3p/*GBAP1* regulatory ceRNA network in human cell lines and in iPSC neurons derived from fibroblasts of PD patients carrying *GBA* mutations, with dopaminergic neurons being used as the control. The results showed that miR22-3p bound to and downregulated *GBA* and *GBAP1* mRNA levels by up to 70%, and that overexpression of the *GBAP1* 3′ UTR sequestered miR-22-3p and thereby caused an increase in *GBA* mRNA and GCase levels. Moreover, multiple out-of-frame isoforms generated from *GBAP1* splicing were identified, and the expression levels of these isoforms were associated with nonsense-mediated mRNA decay, which suggested that *GBAP1* levels and the associated ceRNA effects are modulated by this degradation process (Straniero et al., 2017).

The *ciRS-7* and miR-7 ceRNA machinery mentioned in the preceding section could also affect PD pathogenesis. This is because *α-synuclein* mRNA is one of the targets of miR-7, which is involved in PD pathogenesis (Junn et al., 2009). miR-7 suppresses endogenous *α-synuclein* mRNA levels when transfected in a human cell line, but this effect is counteracted by the overexpression of *ciRS-7*, which suggests a ceRNA regulatory pattern (Hansen et al., 2013). Furthermore, *ciRS-7* is degraded through Ago2-mediated cleavage in a miR-671-dependent manner (Hansen et al., 2011), which indicates that *ciRS-7* potentially transports miR-7 as cargo, which is released by miR-671. Deregulation of such ceRNA networks might contribute to the progression of neurodegeneration. Moreover, several lines of evidence have indicated that miR-7 regulates multiple signaling pathways in cancer pathogenesis by acting on regulatory factors such as epidermal growth factor receptor, insulin receptor substrate (IRS)-1, IRS-2, p21-activated kinase-1, and Raf1 (Reddy et al., 2008; Webster et al., 2009); these findings suggest that ciRS-7 can potentially regulate these pathways in neurological diseases (Shao and Chen, 2016). However, additional experimental data are necessary to validate this hypothesis.

Further evidence of ceRNA regulation is emerging from PD pathological studies. For example, Liu et al. reported that in an MPTP-induced PD mouse model and in MPP^+^-exposed SH-SY5Y cells, the lncRNA *MALAT1* is upregulated, but miR-124 is downregulated (Liu et al., 2017). MiR-124, a brain-enriched miRNA, has been demonstrated to be neuroprotective in certain CNS diseases (Ponomarev et al., 2011), as well as to regulate apoptosis and autophagy in the MPTP model of PD by targeting *Bim* (Wang et al., 2016). Notably, *MALAT1* knockdown was found to attenuate the apoptosis of dopamine neurons in the MPTP-induced PD mouse model and miR-124 overexpression countered this effect, which suggests that *MALAT1* induces apoptosis in the PD model by sponging endogenous miR-124 (Liu et al., 2017). However, further investigation is required to identify the downstream factors that function in this ceRNA regulation.

### Spinocerebellar Ataxia Type 7 (SCA7)

), and by degeneration of the retinal macula, which gradually leads to blindness (Gouw et al., 1994). SCA7 results from an in-frame CAG trinucleotide-repeat expansion in the first coding exon of *ATXN7*, the gene encoding ataxin type 7 (David et al., 1997); this leads to polyglutamine (polyQ)-tract expansion in the translated protein, the formation of protein aggregates, and decreased protein activity. ATXN7 is an essential component of mammalian STAGA multiprotein complex 5, which facilitates the transcriptional activation of multiple loci through its chromatin-remodeling activity (Holmberg et al., 1998).

Numerous ncRNAs exhibit tissue- and cell-type-specific expression patterns (Eddy, 2001), which indicates that ncRNA-directed ceRNA regulation might occur in a localized manner (Taulli et al., 2013). Tan and coworkers presented a striking example of this by showing that ceRNA regulatory loops contribute to tissue specificity in diseases (Tan et al., 2014). According to Tan et al. (2014) under the aforementioned mechanism, the expression pattern of a conserved retropseudogene, *lnc-SCA7* (assigned named *ATXN7L3B*), is significantly correlated with that of *ATXN*7 in adult tissues and in the postnatal CNS across human and mouse. Moreover, an *lnc-SCA7*/miR-124/*ATXN7* ceRNA network was identified, and STAGA was found to be required for the initiation of the transcription of miR-124, which mediates the posttranscriptional crosstalk between *lnc-SCA7* and *ATXN7* mRNA and thus creates a loop. In SCA7, *ATXN7* mutations disrupt this regulatory loop, leading to a neuron-specific increase in ATXN7 expression. Furthermore, in mice, this increase in expression is most prominent in the retina and the cerebellum, which are recognized as SCA7 disease-relevant tissues (Tan et al., 2014; Bayoumi et al., 2016; Salta and De Strooper, 2017).

Thus far, ceRNA investigations in NDDs have mainly focused on AD and PD, and, as discussed above, a few cases of ceRNA regulation have also been reported in SCA7 (**Table 1**). However, in HD and ALS, ceRNA regulation remains undiscovered.

**Table 1:** Competing endogenous RNA studies in neurodegenerative disorders.

ceRNA pathways					
Disease	ceRNA	miRNA	Target RNA	Mechanism	Reference
AD	*BACE1-AS*	miR-485-5p	*BACE1*	Stabilizing *BACE1* mRNA; competing miRNA	Faghihi et al., 2008
	*ciRS-7*	miR-7	*CDR1 UBE2A*	Stabilizing *CDR1* mRNA; competing miRNA; miR-7 sponge	Hansen et al., 2011, 2013; Memczak et al., 2013; Zhao et al., 2016; Salta and De Strooper, 2017; Shi et al., 2017
	*MIAT*	miR-150-5p	*VEGF*	Competing miRNA	Jiang et al., 2016
	*Rpph1*	miR-330-5p miR-326-3P	*Cdc42*	Competing miRNA	Cai et al., 2017
PD	*GBAP1*	miR-22-3p	*GBA*	Competing miRNA	Straniero et al., 2017
	*ciRS-7*	miR-7	*-*	miR-7 sponge	Junn et al., 2009
	*MALAT1*	miR-124	*-*	Competing miRNA	Liu et al., 2017
SCA7	*lnc-SCA7*	miR-124	*ATXN7*	Competing miRNA; indirect control by *ATXN7* of miR-124 transcription initiation; formation of negative-feedback loop	Tan et al., 2014
**ceRNA networks**					
**Disease**	**Mechanism**		**Disease model**		**Reference**

AD	lncRNA–miRNA–mRNA		APP/PSEN1 E9(FAD) mouse model		Cai et al., 2017
	lncRNA–miRNA–mRNA		NFT neurons from entorhinal cortex of mid-stage AD cases		Wang L.K. et al., 2017
	circRNA–miRNA–mRNA		SAMP8 (SAD)/SAMR1		Zhang et al., 2017

## Challenges and Insights

### miRNA and ceRNA Hierarchies in Cross-Regulations

An intriguing question underlying the efforts to decode the complexity of ceRNA regulatory networks is this: Do hierarchies exist between miRNAs and ceRNAs at the level of cross-regulation? (Guil and Esteller, 2015). Accumulating evidence has helped refine the dynamics and constraints in ceRNA regulation (Tay et al., 2014), revealing that various factors could contribute toward creating miRNA and ceRNA hierarchies; these factors can be summarized as follows: miRNA target-site efficacy; shared MRE abundance; miRNA/ceRNA expression level and subcellular localization (Salmena et al., 2011; Tay et al., 2014); miRNA:target ratio; competition between rate-limiting molecules, such as Ago (Figliuzzi et al., 2013); and advanced ceRNA hierarchy strategies. Here, we discuss recent studies indicating the existence of these hierarchies.

**FIGURE 2:**
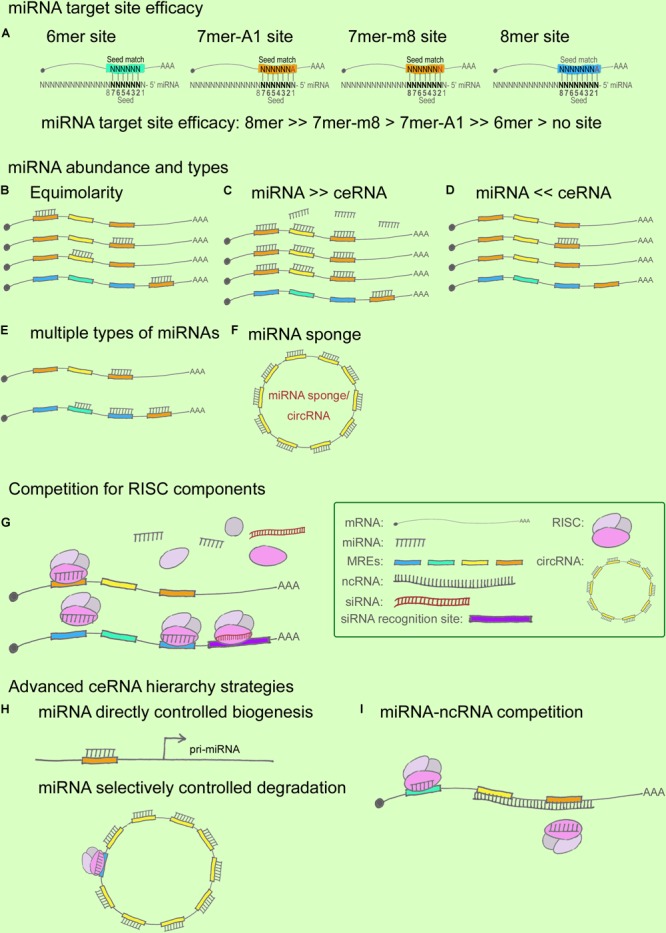
miRNA and ceRNA hierarchies in cross-regulations. **(A)** miRNA target-site efficacy, from strong to weak, follows this order: 8mer > > 7mer-m8 > 7mer-A1 > > 6mer > no site. In this cartoon, the seed region flanking the sequence position 2–7 is shown in black. In a 6mer site, a 6-nt sequence match exists between the seed region of the miRNA and the target mRNA. Similarly, a 7mer-A1 site contains a 7-nt seed match with an A at position 1 on the target, whereas a 7mer-m8 site contains a paired N at position 8. An 8mer site contains an 8-nt seed match with both an A at position 1 and a paired N at position 8. **(B–F)** miRNA/ceRNA abundance and MRE types. ceRNA cross-regulation reaches optimal effectiveness when miRNA and ceRNA targets are present in almost equimolar amounts **(B)**; ceRNA regulation is negligible when the abundance of the miRNA is substantially greater than that of the ceRNA targets **(C)**, and vice versa **(D)**; ceRNA regulatory power is increased when a ceRNA targets ≥ 2 miRNAs whose downstream ceRNA targets are involved in producing the same effect in a pathway **(E)**; ceRNA regulation is strongly affected when an miRNA sponge or a circRNA contains multiple seed-sequence matching sites for an miRNA **(F)**. **(G)** Competition for RISC components. Ago abundance is one of the rate-limiting factors in ceRNA regulation, and miRNAs and siRNAs compete for RISC and RISC components such as Ago and Dicer. **(H,I)** Advanced ceRNA hierarchy strategies. Certain miRNAs directly control the biogenesis of other miRNAs and ncRNAs **(H)**; for example, nuclear miR-709 targets pri-miR-15a/16-1, and the *ATXN7*-miR-124 loop controls pri-miR-124 transcription. miRNAs can also selectively control degradation **(H)**; for instance, *ciRS-7* can bind to as many as 74 miR-7s without degradation being triggered, whereas miR-671 targets *ciRS-7* for degradation. ncRNAs can compete with miRNAs by masking the miRNA seed regions on a ceRNA target, as well as by stabilizing the ceRNA target **(I)**.

First, miRNA target-site efficacy: As discussed in relation to the miRNA functional machinery in the previous section, miRNAs have long been considered to play the role of “fine-tuners” in posttranscriptional regulation (Figliuzzi et al., 2013). miRNA target-site efficacy is determined by multiple factors: One, a favorable sequence context around the miRNA-binding site is critical, where the seed-sequence length and specific binding regions are the key determinants (**Figure 2A**). The relative efficacy of miRNA target sites has been summarized as 8mer >> 7mer-m8 > 7mer-A1 >> 6mer > no site (Bartel, 2009). Specifically, 7mer sites were found to be 50% as effective as 8mer sites and 6mer sites were 20% as effective in contributing to target abundance (Denzler et al., 2016). In terms of binding locations: 7mer-m8 sites in 3′ UTRs > 7mer-m8 sites in the central region of long 3′ UTRs > 8mer in the path of the ribosome (Bartel, 2009). Two, the presence of multiple and cooperatively spaced binding sites affects efficacy (Denzler et al., 2016). Three, the local AU content and the supplemental 3′ paring influence efficacy (Bartel, 2009). Lastly, additional factors to consider are successful recruitment by the Ago-directed RISC (Bosson et al., 2014), and the secondary structure of RNA, which might contribute to the miRNA binding dynamics.

An increase in shared MRE types and their abundance can fine-tune ceRNA crosstalk (**Figures 2B–F**), which reaches optimal effectiveness when miRNAs and ceRNAs are present at almost equimolar amounts (Ala et al., 2013; Figliuzzi et al., 2013) (**Figures 2B–D**). Specifically, it has been hypothesized that when miRNAs are highly abundant, ceRNA expression levels would exert little effect on ceRNA regulation, and, conversely, that under a low abundance of miRNAs, ceRNA regulation would exert little effect because a minimal number of targets would be bound in the given system (Wee et al., 2012; Tay et al., 2014). Recent studies have provided sufficient statistical evidence indicating how MRE abundance can alter ceRNA crosstalk. Ala et al. (2013) reported that ceRNA activity *in silico* can achieve optimal effectiveness within a certain expression window for both miRNAs and ceRNAs, and further that the indirect crosstalk in a ceRNA network can amplify the ceRNA signal. Moreover, Figliuzzi et al. (2013) described ceRNAs at steady state by using a minimal rate equation-based model. These two studies have demonstrated that the abundance of miRNAs/ceRNAs and the shared MREs, as well as the indirect crosstalk, are key factors for regulating ceRNA fluctuation (Tay et al., 2014). Recent ceRNA research has further shown that besides MRE abundance, the shared types of MRE affect ceRNA crosstalk: the higher the number of MRE types shared between ceRNAs, the higher the efficiency of ceRNA regulation (Salmena et al., 2011; Ala et al., 2013), particularly when a ceRNA targets two or more miRNAs whose downstream ceRNA targets are involved in the same signaling pathway (Cai et al., 2017) (**Figure 2E**). For instance, ciRS-7 functions as a powerful circRNA sponge by providing 74 seed-sequence matches for miR-7; ciRS-7 has thus emerged as a key regulator and potential therapeutic target in NDDs such as AD and PD (Salta and De Strooper, 2017). However, most of the reported mammalian miRNA sponges contain only one or two binding sites for the same miRNA, which limits their potency (Poliseno et al., 2010; Memczak et al., 2013). Moreover, the expression levels of both miRNAs and ceRNAs at distinct subcellular locations represent another crucial determinant for a refined ceRNA-crosstalk hierarchy, because multiple lines of evidence have indicated that numerous ncRNAs are expressed in spatiotemporally specific patterns (Eddy, 2001).

Ago, the catalytic component of RISC, is a rate-limiting factor (**Figure 2G**): Loinger and coworkers found that Ago abundance is a rate-limiting determinant of ceRNA crosstalk by analyzing mathematical models, which revealed that the lower the amount of Ago, the stronger the competition (Loinger et al., 2012; Tay et al., 2014). In a paradigm involving multiple ceRNA players, downregulation of the targets of the trigger miRNA was found to be a direct effect of the miRNA transfection, whereas the upregulation of the targets of perturbed miRNAs was an indirect effect. The competition for a limited supply of Ago underlies both scenarios, where the direct effect appears to be stronger than the indirect effect (Loinger et al., 2012). Moreover, siRNAs were considered in the Ago competition, because both miRNA and siRNA pathways share common components (Filipowicz et al., 2005; Gregory et al., 2005), and saturation of the endogenous RNAi machinery with siRNAs results in the perturbation of endogenous miRNA function (Khan et al., 2009; Loinger et al., 2012). Recently, RISC has been shown to act as a multi-turnover enzyme and catalyze multiple rounds of RNA cleavage (Hutvagner and Zamore, 2002; Haley and Zamore, 2004; Filipowicz, 2005; Filipowicz et al., 2005), although the recycling mechanism remains elusive. Following the same logic, the competition involving other RISC components, such as Dicer, might represent a part of this story (Loinger et al., 2012). These findings have helped broaden the paradigm presented by the widely recognized and simplified model including one miRNA and 2 ceRNAs.

Advanced ceRNA hierarchies have now started being identified (**Figures 2H,I**). First, accruing evidence indicates that nuclear miRNAs can directly regulate the biogenesis of other miRNAs and lncRNAs (Hansen et al., 2011; Tang et al., 2012). This notion is supported by several findings, such as the following: certain mature miRNAs can re-enter the nucleus (Meister et al., 2004; Hwang et al., 2007); active miRNA effectors, including Ago proteins, are localized in the nucleus (Robb et al., 2005); exportin-1 allows nucleocytoplasmic shuttling of mature miRNAs in a complex containing Ago proteins (Castanotto et al., 2009), which suggests that exportin-1 might serve as a pore for Ago translocation between the cytoplasm and the nucleus (Zisoulis et al., 2012); and Ago2 is transported into the nucleus by importin-8 in human cells (Weinmann et al., 2009). One of the examples relevant in the context of the CNS is that of the circRNA *CDR1* mentioned earlier; this circRNA is complementary to a nucleus-enriched miRNA, miR-671, which directs the Ago2-mediated cleavage of a *CDR1* antisense transcript (Hansen et al., 2011; Chen et al., 2012; Liang et al., 2013) (**Figure 2H**). Another CNS-related example (also noted above) is the *lnc-SCA7*/miR-124/*ATXN7* auto-feedback loop that regulates ceRNA transcription (Tan et al., 2014). According to these functional models, miRNAs control ncRNA homeostasis by forming tightly regulated ceRNA hierarchies (Chen et al., 2012). Second, in their study of *BACE1-AS*, Faghihi and colleagues sought to determine the extent to which mRNA stabilization by complementary ncRNA represents a general theme in the human genome; the investigators selected a set of evolutionarily conserved sense-antisense pairs in human and mouse genomes and predicted the miRNA-binding sites within pairing (sense-antisense overlapping) regions and non-pairing (non-overlapping) regions (Faghihi et al., 2010). The results showed that miRNA-binding sites were enriched in the non-overlapping regions of sense-antisense pairs, which suggests that overlapping regions between sense and antisense RNA transcripts are functional regulatory elements, and that the formation of a sense-antisense RNA duplex might prevent miRNA binding. Faghihi and colleagues suggested that a selection might occur to avoid a clash of two regulatory elements within a specific region (Faghihi et al., 2010); the findings imply that a perfect match between RNAs facilitates the formation of RNA duplexes, which leads to RNA stabilization instead of degradation, whereas the generation of miRNA-mRNA complexes more likely leads to RNA degradation. The phenomena observed in the study suggest an intriguing ceRNA hierarchy involved in evolutionary selection, but this remains to be comprehensively investigated. Third, mRNA 3′ UTR shortening by alternative polyadenylation is recently reported in disrupting ceRNA regulation in breast cancer (Park et al., 2018), which provides intriguing insights of the participation of mRNA 3′ UTR dynamics in ceRNA hierarchies in NDDs.

As we have discussed in this session, miRNA affinities, MRE types and abundance, RNA concentrations in specific cellular locations, amounts of Ago and other RISC components, and other advanced evolutionary strategies collectively contribute toward generating ceRNA hierarchies in the regulation of RNA homeostasis.

### Balance Between Destabilization and Stable Binding

An intriguing phenomenon is that ceRNA regulation can be symmetrical, where 2 ceRNAs reciprocally regulate each other (Tan et al., 2014; Straniero et al., 2017), or asymmetrical, where one ceRNA regulates the other but not vice versa (Hansen et al., 2013; Tay et al., 2014). This raises the question as to what the molecular determinants are that indicate mRNA/ncRNA destabilization versus stable binding (i.e., translation inhibition in the case of a coding ceRNA) when considering ncRNA–miRNA or mRNA–miRNA interactions (Guil and Esteller, 2015). Numerous lines of evidence support the view that miRNAs regulate target mRNAs through a combination of translational repression (Wightman et al., 1993; Olsen and Ambros, 1999; Seggerson et al., 2002) and mRNA destabilization (Bagga et al., 2005; Lim et al., 2005). Whereas inhibition of translational initiation is a rate-limiting step in translational repression (Pillai et al., 2007), mRNA destabilization (in other words, mRNA degradation) is a consequence of miRNA-mediated deadenylation of target mRNAs (Behm-Ansmant et al., 2006; Giraldez et al., 2006; Wu et al., 2006), which leads to the decapping and 5′–3′ decay of these mRNAs (Rehwinkel et al., 2005; Behm-Ansmant et al., 2006; Chen et al., 2009). Although both translational repression and mRNA decay can result in reduced protein synthesis, the mechanism underlying repression holds critical biological implications. When repression occurs through translation inhibition, rapid recovery of translation to the original level can occur in the absence of new transcription. By contrast, the reversal of miRNA-mediated repression requires new transcription, because mRNA decay constitutes the major mode of repression (Eichhorn et al., 2014). Three papers published in 2012 provided insights into the dynamics of miRNA-mediated repression in fly, zebrafish, and human cells (Bazzini et al., 2012; Bethune et al., 2012; Djuranovic et al., 2012). Furthermore, Eichhorn and colleagues made valuable contributions toward uncovering the ratio of miRNA-mediated mRNA destabilization and translational inhibition in mammalian cells (Eichhorn et al., 2014). As compared to translational repression, mRNA destabilization becomes detectable after a longer lag, presumably because more time is necessary for mRNA decay than inhibition of translation initiation. However, this lag period is brief, and destabilization soon dominates. Eichhorn et al. (2014) showed that mRNA destabilization explains 66–90% of the miRNA-mediated repression observed at steady state. These findings strongly support a model wherein miRNAs actively recruit the deadenylation machinery, and in this model, mRNA deadenylation, decapping, and decay constitute the major mode of miRNA-mediated repression of endogenous targets in mammalian cells (Eichhorn et al., 2014).

The analysis discussed above did not include certain outliers, such as a single-gene result, where the miRNA-dependent change is observed in the level of the protein (by immunoblotting) but not mRNA (by using quantitative RT-PCR) (Eichhorn et al., 2014). The miR-7-sponge *ciRS-7* is one such example: When circRNA- or linear-RNA-producing vectors of *ciRS-7* were coexpressed with miR-7 or miR-769 expression vectors, *ciRS-7* levels were unaffected, but the linear construct displayed miR-7 sensitivity and was lowered by >40% ± 12%, presumably because of miRNA-mediated activation of deadenylation and subsequent exonucleolytic degradation. This finding suggests that the target sites in *ciRS-7* do not support endocleavage by miR-7, and that *ciRS-7* is resistant to the conventional miRNA-mediated destabilization of mRNA (Hansen et al., 2013).

The polyadenylation of mRNA occurs dynamically under distinct cellular conditions and at different developmental stages (Weill et al., 2012) and regulates mRNA stability, translation, and transportation (Di Giammartino et al., 2011). Intriguingly, mRNA deadenylation does not unfailingly lead to mRNA degradation: Deadenylated mRNAs can be translationally silent but stable and can be reactivated by cytoplasmic polyadenylation, and this mechanism allows gene expression to be resumed even in the absence of transcription (Mendez and Richter, 2001). For example, in mammalian neurons, synaptically localized mRNAs, such as CaMKII mRNA, are repressed and transported to postsynaptic densities, and then reactivated for local translation by cytoplasmic polyadenylation upon synaptic stimulation (Weill et al., 2012). Moreover, CPEB1 is actively involved in these processes (Weill et al., 2012). Recent studies have indicated that cell state is one of the main determinants of the fate of miRNA-mRNA binding (Subtelny et al., 2014). Subtelny and colleagues showed that poly(A) tail lengths were strongly linked to translational efficiencies in the early developmental stages of zebrafish and frog embryos, and this association was diminished at gastrulation and absent in non-embryonic stages (Subtelny et al., 2014). These findings indicate that the translational control mechanism features a rapid developmental switch and explain why the predominant effect of miRNA-mediated deadenylation concurrently shifts from translational repression to mRNA destabilization (Subtelny et al., 2014). Subtelny and colleagues concluded that if the cell is in the oocyte or early embryo stage, miRNA-mediated deadenylation causes diminished translation, whereas later in development, it primarily causes mRNA destabilization coupled with a slight additional effect on translation (Subtelny et al., 2014).

Despite the fundamental differences between the properties of ncRNAs and mRNAs, linear ncRNAs in the cytoplasm are assumed to be regulated in the same manner as mRNAs (Subtelny et al., 2014). circRNAs are not expected to undergo miRNA-mediated repression, because they lack a poly(A) tail, although recent findings have shown that *ciRS-7* is destabilized by miR-7 (Kleaveland et al., 2018). However, other underlying determinants of the tradeoff between miRNA-mediated ncRNA destabilization and stable binding remain unknown (Memczak et al., 2013). Furthermore, target-RNA-directed miRNA degradation (TDMD) (Ameres et al., 2010) contributes to an additional layer of complexity of ceRNA hierarchies. Specifically, most miRNAs are stable (Guo et al., 2015), which can be attributed to Ago binding protecting against degradation by exonucleases (Elkayam et al., 2012), but under a few conditions, such as in response to neuronal excitation (Krol et al., 2010), certain miRNAs are destabilized, probably through the TDMD induced by extensive miRNA-target complementarity (Denzler et al., 2016; Kleaveland et al., 2018).

### The ceRNA Debate: Providing Insights in the Context of Disease and Therapeutic Applications

Shortly after the ceRNA hypothesis was proposed in 2011 (Salmena et al., 2011), ample experimental evidence supporting and disputing the hypothesis emerged (Broderick and Zamore, 2014). The ceRNA debate has mainly addressed the refractory ceRNA regulation that occurs under normal physiological concentrations of miRNAs and ceRNAs by using cell lines, *in vivo* mouse models, and mathematical models (Mullokandov et al., 2012; Denzler et al., 2014; Jens and Rajewsky, 2015). According to the ceRNA hypothesis, miRNAs and their binding sites must be of similar abundance, with the regulation being optimally effective when miRNAs and their ceRNA targets are near equimolarity (Ala et al., 2013; Bosia et al., 2013; Figliuzzi et al., 2013), and ceRNA transcripts compete with each other to bind to a limited pool of miRNAs, and this regulates the transcript level and protein translation of the targeted ceRNA. Adding to the ceRNA debate, Denzler and colleagues have proposed a new model based on *in vivo* experiments wherein target sites exceeded miRNAs in a physiological context, a scenario in which almost all the miRNA is in a complex with RISC and no free miRNA pool is available for competition (Broderick and Zamore, 2014; Denzler et al., 2014, 2016). The example used for testing this model was miR-122 in hepatocytes, and the release of endogenous-target repression began to be observed only when the number of added miR-122 target sites reached a threshold of 1.5 × 10^5^/cell, which exceeds the physiological levels of endogenous targets in health and disease (Denzler et al., 2014). Denzler and colleagues stated that under physiological conditions, the change caused by ceRNA perturbation is probably too small to be detected and to produce biological consequences; however, the investigators also noted that this did not imply a complete absence of any molecular consequence of changing the level of an endogenous target and that it remained possible that highly abundant and regulated ceRNAs might substantially contribute to the pool of transcriptome binding sites (Denzler et al., 2014). Mullokandov and coworkers showed that only the most abundant miRNAs in a cell mediate target suppression, and that >60% of the detected miRNAs exhibited no discernible activity (Mullokandov et al., 2012). A similar idea was also favored in a recent study by Jens and Rajewsky (2015).

**FIGURE 3:**
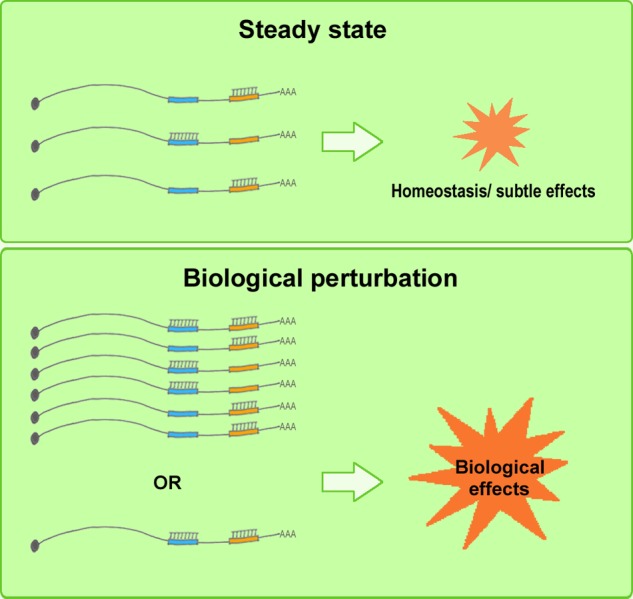
Elevated biological effects of ceRNAs under biological perturbation. Under steady-state conditions **(upper)**, well-regulated, subtle perturbation of miRNA and ceRNA expression levels is not highly likely to induce detectable changes in target RNA levels or marked biological alterations. Under biological perturbation **(lower)**, miRNA and ceRNA levels increase or decrease substantially in certain physiological stages, such as cell differentiation or tumorigenesis, and in certain cell types and subcellular locations, and this tends to maximize the biological effectiveness and significance of ceRNA regulation.

Although a considerable amount of experimental evidence has been published supporting ceRNA regulation, the ceRNA debate, in particular, has helped enhance our understanding of the inherent nature of the complex RNA regulatory network *in vivo*. A re-evaluation of the ceRNA mechanism reveals the following: Whereas a few miRNA mutants produce dramatic phenotypes, most merely fine-tune mRNA expression. More than half of all human mRNAs contain predicted miRNA-target sites, and, collectively, miRNAs regulate nearly all developmental pathways (Friedman et al., 2009; Broderick and Zamore, 2014). This raises the following question: Why do cells contain low-abundance miRNAs when only the most abundant miRNAs appear to be functionally involved in regulation? According to Broderick and Zamore, the miRNA language could be simple; the miRNAs present in limited amounts in one cell type or developmental stage are probably abundant in other cell types/developmental stages or under altered physiological conditions (Broderick and Zamore, 2014). During both normal cell differentiation and malignant transformation, the expression levels of coding RNAs and ncRNAs can change drastically (Rhodes and Chinnaiyan, 2005; Lujambio and Lowe, 2012). Under such biological conditions, changes in miRNA and ceRNA levels could make the system highly amenable to ceRNA-mediated gene regulation (Denzler et al., 2014). Moreover, Mukherji and colleagues performed single-cell measurements to monitor the protein level of a target gene in the presence and absence of miRNA-mediated regulation (Mukherji et al., 2011), and the results showed that although the average level of repression was modest, the repression among individual cells varied substantially. Specifically, regulation by miRNAs established a threshold level of target mRNA, and below this threshold, protein production was highly repressed, whereas near the threshold, protein expression responded sensitively to target mRNA input; this finding suggests that an miRNA can function as both a switch and a fine-tuner of gene expression (Mukherji et al., 2011). These results agree with the conclusions related to ceRNA hierarchy. Furthermore, certain circRNAs (such as *ciRS-7*) contain numerous binding sites for miRNAs (such as miR-7), which indicates the existence of potent endogenous RNA sponges (Lukiw, 2013; Jens and Rajewsky, 2015). Collectively, these findings support the following view: miRNAs act as fine-tuning regulators and maintain RNA homeostasis in a physiological context, where well-regulated, subtle perturbations of the expression levels of miRNAs and ceRNAs are not highly likely to produce either detectable changes in target RNA levels or notable biological alterations; these changes are observed only when the miRNA/ceRNA levels increase or decrease drastically in certain physiological states, such as cell differentiation or tumorigenesis, and in certain cell types and subcellular locations, and these circumstances maximize their biological effectiveness and significance (**Figure 3**), thus providing insights into potential molecular targets and therapeutic strategies for rational drug design and clinical applications (Bayoumi et al., 2016).

The experimental strategies currently used for studying ceRNA mechanisms could be improved. Large amounts of data from ceRNA studies are typically acquired *in silico* or *in vitro* or from experimental systems in which rapidly dividing cells are transfected with synthetic miRNAs; these approaches do not represent the most satisfactory methods of representing the *in vivo* levels of endogenous RNAs (Arvey et al., 2010; Garcia et al., 2011; Tay et al., 2011; Denzler et al., 2014). Notably, Jens and Rajewsky emphasized that neither the overexpression of a putative ceRNA nor its knockdown by using an siRNA provides a direct test for the hypothesis regarding interaction through competition for miRNAs (Jens and Rajewsky, 2015). When overexpressed, almost any RNA could function as a strong miRNA sponge because of being present in unphysiologically high copy numbers, whereas in the case of siRNA-mediated knockdown, high expression of the siRNA can overwhelm the miRNA machinery by competing for RISC components (Filipowicz et al., 2005; Khan et al., 2009; Jens and Rajewsky, 2015). In such cases, target-site protectors (Choi et al., 2007) or mutagenesis of the potential MREs by using the CRISPR-Cas system (Cong et al., 2013; Mali et al., 2013) could be used in attempts to closely mimic the ceRNA function in an endogenous context. Moreover, in a valid rescue experiment, a minimal construct bearing only the presumed competing binding sites would be expressed at endogenous levels (Jens and Rajewsky, 2015). Although canonical CRISPR-Cas9 knockout might not be appropriate for ncRNAs because of the lack of ORFs, CRISPR interference provides an alternative method to achieve sequence-specific control of ncRNA expression (Larson et al., 2013). Furthermore, technologies such as RNA sequencing in situ offer the possibility of quantifying transcript levels at distinct subcellular locations (Lee et al., 2014).

## Perspectives and Concluding Remarks

The RNA-world model has grown rapidly over the past two decades. Once regarded as evolutionary junk, ncRNAs are now recognized as key actors on the biological stage that play critical roles in regulating diverse biological processes and disease pathogenesis (Eddy, 2001). The emergence of the ceRNA hypothesis not only helped explain the occurrence of a large number of miRNA seed sequences in mRNAs and in other ncRNAs during evolution: the hypothesis also provided valuable insights into the previously unrecognized manner in which highly complex RNA networks are orchestrated. As numerous ncRNAs were identified using next-generation sequencing techniques, several ncRNAs were found to function in ceRNA loops in disease contexts (Taulli et al., 2013; Kartha and Subramanian, 2014). The earliest and the majority of ceRNA regulations were reported in cancers (Tay et al., 2011; Kartha and Subramanian, 2014), but increasing numbers of ceRNA loops (Faghihi et al., 2008; Modarresi et al., 2011; Tan et al., 2014; Shi et al., 2017; Straniero et al., 2017) and networks (Cai et al., 2017; Wang L.K. et al., 2017; Zhang et al., 2017) were found to regulate NDDs (Johnson et al., 2012; Salta and De Strooper, 2017). As summarized in this review, the list of ceRNA studies in NDDs is heretofore small. Most ceRNA regulations are reported in AD models, while some others are reported in PD and SCA7. One potential reason for more ceRNA regulations being reported in cancer than NDDs is that the fold-change in the expression of differentially expressed genes in cancer is typically higher than that in NDDs. Nonetheless, given the rapid pace at which previously unknown RNAs such as circRNAs have been discovered, we can expect the identification in the near future of additional currently unrecognized ceRNA regulatory loops and circuitries.

Because of the complexity involved in ceRNA regulation, both challenges and insights have emerged from the investigations into this RNA regulatory mechanism, and several questions regarding the mechanism remain unanswered. In this review, we have attempted to address three key questions posed by Guil and Esteller (2015). The most appealing proposition related to the regulation is miRNA/ceRNA hierarchies in cross-regulation. When vast numbers of miRNAs and ceRNAs are present in a specific subcellular location, factors such as miRNA target-site efficacy, MRE types and abundance, and competition for Ago and other RISC components influence the assembly of organized and dynamic hierarchies in ceRNA cross-regulation. Furthermore, identification of other intricate strategies, such as the involvement of nuclear miRNAs in directly controlling the biogenesis of other miRNAs and lncRNAs, has extended our understanding of the ceRNA hierarchy (Chen et al., 2012). However, the rules that potentially govern the establishment of ceRNA hierarchies must be further identified, such as by determining whether an RNA secondary structure can dynamically regulate ceRNA crosstalk.

One of the most critical questions in ceRNA crosstalk is this: What determines the fate of the ceRNAs that bind to miRNAs and undergo destabilization or stable binding? (Guil and Esteller, 2015). Regarding miRNA–mRNA interactions, previous studies have uncovered the temporal dynamics underlying mRNA destabilization and translational inhibition. Specifically, translational inhibition dominates first because of being the relatively faster biological response, but mRNA destabilization dominates after an initial lag (Eichhorn et al., 2014). Given that ncRNAs generate no proteins, i.e., do not undergo translation, what could be the fate of the ncRNAs in an ncRNA–miRNA–ncRNA or ncRNA–miRNA–mRNA loop? It is crucial to elucidate the fate of ceRNAs and miRNAs in a ceRNA regulatory system in both health and disease, because the balance between destabilization and stable binding will be closely related to miRNA and ceRNA recycling and the effective ceRNA concentration.

The question that remains most debated in relation to ceRNA regulation is whether this regulation is effective in physiological contexts. Most studies challenging ceRNA roles conclude that ceRNA regulation is unlikely to produce biologically significant effects under physiological concentrations of the RNAs; however, these studies cannot exclude the possibility of either the existence of potent miRNA sponges or the marked upregulation or downregulation of miRNAs and ceRNAs at specific developmental stages or subcellular locations (Denzler et al., 2014). Numerous studies have reported that ceRNA machineries operate in various diseases and that ceRNA expression patterns vary with varying tissue, cellular, and subcellular conditions, and these studies have provided new insights that can facilitate the design of ceRNA-mechanism-based therapeutic applications for manipulating specific developmental stages and disease pathogeneses by using synthetic sequence-specific oligonucleotides (Khvorova and Wolfson, 2012). Because the impact of miRNA perturbation might be large but concurrently also poorly predictable, the use of target-site protectors against specific MREs appearing on ceRNAs might represent a favorable solution (Choi et al., 2007; Khvorova and Wolfson, 2012). Moreover, experimental approaches in ceRNA studies can be improved by employing strategies such as using target-site protectors for MREs, knocking in MRE mutations by using the CRISPR-Cas9 system, or controlling gene expression by using CRISPR interference. In summary, as we continue to face new challenges in the study of ceRNA regulation and obtain new insights into the mechanistic underpinnings of this regulation, our understanding of the nature of ceRNA crosstalk is also continuing to improve; this enhanced understanding will not only help guide our future exploration of the mysteries of the modern RNA world, but also enable us to develop ceRNA-based applications suitable for both biological studies and therapeutic purposes.

## Author Contributions

YC and JW wrote the manuscript, and have approved submission of this work.

## Conflict of Interest Statement

The authors declare that the research was conducted in the absence of any commercial or financial relationships that could be construed as a potential conflict of interest.

## Abbreviations

Aβ: amyloid-β peptide
AD: Alzheimer’s disease
Ago: Argonaute
CDR1: cerebellar degeneration-related protein 1
ceRNA: competing endogenous RNA
circRNA: circular RNA
*ciRS-7*: circular RNA sponge for miR-7
FAD: familial Alzheimer’s disease
lncRNA: long non-coding RNA
miRNA: microRNA
MREs: miRNA-response elements
ncRNA: non-coding RNA
NDD: neurodegenerative disorders
ORF: open reading frame
PD: Parkinson’s disease
RISC: RNA induced silencing complex
SAD: sporadic Alzheimer’s disease
SCA7: Spinocerebellar ataxia type 7
siRNA: small interfering RNA
